# Dual function of *Desmidorchis retrospiciens*-derived gold nanoparticles as antibacterial and osteoinductive agent for treating osteomyelitis

**DOI:** 10.3389/fmicb.2025.1633245

**Published:** 2025-08-04

**Authors:** Basem M. Abdallah, Peramaiyan Rajendran, Enas M. Ali

**Affiliations:** ^1^Department of Biological Sciences, College of Science, King Faisal University, Al-Ahsa, Saudi Arabia; ^2^King Salman Center for Disability Research, Riyadh, Saudi Arabia

**Keywords:** gold nanoparticles, disability, osteomyelitis, osteoinductive, antibacterial, MRSA

## Abstract

**Introduction:**

Osteomyelitis is a chronic inflammation of bone due to pathogenic infection mainly by methicillin-resistant *Staphylococcus aureus* (MRSA). Osteomyelitis can cause severe bone damage and pathologic fractures, which subsequently result in a high physical disability rate. Thus, a therapeutic agent with antibacterial and bone regenerative activities can be effective for managing osteomyelitis.

**Methods:**

We phyto-fabricated gold nanoparticles using plant extract from *Desmidorchis retrospiciens* (DR-AuNPs) and examined their antibacterial and osteoinductive activities. The biosynthesis of DR-AuNPs was confirmed with several characterization techniques, including scanning and transmission electron microscopy, X-ray diffraction (XRD), energy-dispersive X-ray analysis (EDX), and Fourier transform infrared spectroscopy (FTIR). Biofilm assays and reactive oxygen species (ROS) analysis were used to examine the antibacterial effect of DR-AuNPs. *In vitro* and *ex vivo* cell biology assays were used to study the osteoinductive effect of DR-AuNPs.

**Results:**

DR-AuNPs displayed substantial antibacterial activity against MRSA-induced osteomyelitis by increasing ROS production, membrane leakage, membrane permeability, and disruption of surface structure in the MRSA. Several anti-biofilm assays and electron microscopic studies have demonstrated the efficacy of DR-AuNPs in significantly inhibiting the formation of MRSA biofilm. Interestingly, DR-AuNPs were also found to promote the lineage commitment of bone marrow-derived mesenchymal stem cells (BMSCs) into osteoblasts *in vitro* and to enhance their capacity for ectopic bone formation *in vivo* without causing any cell toxicity.

**Discussion:**

Our data provide green DR-AuNPs with dual antibacterial and osteoinductive activities, making them a promising bioactive agent for managing osteomyelitis.

## Introduction

Infectious osteomyelitis is an inflammation of the bone and bone marrow, primarily caused by bacterial infection. Several bacteria can cause chronic osteomyelitis, including *Staphylococcus aureus, Pseudomonas aeruginosa, Propionibacterium* species, *Enterobacteriaceae* species, *Salmonella* species, and *Streptococcus pneumoniae* (Hatzenbuehler and Pulling, 2011). Gram-positive *S. aureus* accounts for nearly 60% of causative pathogens (Hofstee et al., 2020). Of these, more than 28% are methicillin-resistant *S. aureus* (MRSA) (Silago et al., 2020), making MRSA a common causative pathogen in this type of infection (Turner et al., 2019). Assuming the MRSA antibiotic resistance, “MRSA infections continue to be a major public health distress”, as described by the latest World Health Organization (WHO) report (World Health Organization, 2021). MRSA commonly displays great resistance to co-trimoxazole, ciprofloxacin, and erythromycin (Singh et al., 2017).

The biofilm-mediated nature of osteomyelitis is significant in medical and experimental situations. Numerous biofilm pathogens are uncultivable and display a different phenotype concerning growth rate and antimicrobial resistance (Junka et al., 2017; Wolcott and Ehrlich, 2008). The struggle in eliminating biofilms with common antibiotics partially clarifies why the great success rates of antimicrobial treatment have generally not yet been recognized for orthopedic infections. The establishment of resistant biofilm pathogens reduced the penetration of antimicrobial drugs into bone, and the adverse actions associated with systemic toxicity (Stoodley et al., 2011).

Osteomyelitis can be divided into three main types: hematogenous osteomyelitis caused by infection through the blood circulation, contiguous osteomyelitis caused by spreading infection from adjacent infected tissues, and secondary osteomyelitis caused by infection associated with vascular insufficiency or neuropathy (i.e., patients with diabetic foot infection) (Zapata et al., 2022; Urish and Cassat, 2020). Binding of *S. aureus* to bone extracellular matrix and their internalization into bone cells (osteoblasts) via cell surface fibronectin protein allowed them to avoid attack by the host innate immune effectors and systemic antibiotics (Wen et al., 2020). This pathogenic mechanism can lead to reduction of osteoblast activity and increase inflammation-mediated osteoclast activity and consequently impaired bone homeostasis (Gimza and Cassat, 2021).

Osteomyelitis treatment required surgical intervention in addition to antibiotic therapy. The protocols for the treatment are still complicated due to the heterogeneity of the disease and the inconsistency in diagnostic principles. In this context, systemic or local antibiotics administration remains the primary therapy for osteomyelitis (Zapata et al., 2022; Lima et al., 2014). The reduced rate of osteomyelitis recovery can be attributed to increased antimicrobial resistance and decreased antibiotic tolerance due to bacterial intracellular survival in bone microstructure, which promotes the formation of small colony variants and persisters (Gimza and Cassat, 2021; Abdulrehman et al., 2024). The effective treatment of osteomyelitis should include an antimicrobial agent that can avoid the need for surgical removal of necrotic bone tissues.

Several studies showed the promising use of nanoparticles in the diagnosis and treatment of osteomyelitis (Zapata et al., 2022; Snoddy and Jayasuriya, 2016). Gold nanoparticles (AuNPs) made a significant contribution to various biomedical applications, including drug delivery, biological imaging, diagnosis, and treatment of diseases. This is due to their good biocompatibility, easy synthesis, optical properties, and ability to be functionalized (Khlebtsov et al., 2013; Georgeous et al., 2024). AuNPs have shown a high osteogenic capacity to stimulate osteoblast differentiation *in vitro* and to facilitate bone regeneration *in vivo* in many studies (Babuska et al., 2022). In addition, the use of AuNPs offers a promising approach to managing infections triggered by multidrug-resistant bacteria (Natan and Banin, 2017).

Plant-based nanoparticles offer many advantages over physical or chemically synthesized nanoparticles, including biocompatibility, low toxicity, surface ligand functionalization, and benefits of bioactive phytochemicals as capping agents (Karnwal et al., 2024). The plant extract-mediated AuNPs biosynthesis was extensively investigated, and several plants were used to biosynthesize gold nanoparticles (Khan et al., 2019).

*Desmidorchis retrospiciens* (Ehrenb.) belongs to the family Apocynaceae and is an extensively dispersed succulent taxon found in the dry areas of the world (Awadh Ali et al., 2017). The plant is principally distributed in the semi-desert areas of Saudi Arabia and Africa. Conventionally, the people of Saudi Arabia used *D. retrospiciens* for wound curing; however, no scientific reports demonstrate the wound-healing capacity or antimicrobial action of this plant. Thus, the current study aimed to phyto-fabricate AuNPs using aqueous leaf extract of the wild plant *D. retrospiciens* (DR-AuNPs) for the first time and to establish their antibacterial and osteo-inductive activities. Our results demonstrate the efficiency of biosynthesized DR-AuNPs in inhibiting MRSA-induced osteomyelitis and endorsing the osteogenesis of BMSCs both *in vitro* and *in vivo*.

## Materials and methods

### Collection of the plant material and preparation of leaf extract

*Desmidorchis retrospiciens* leaves were collected in April 2023 from Al-Ahsa-Dammam Road, eastern province, Saudi Arabia, at an altitude of 2515 m with a latitude of 28′45″ N and longitude of 83′23″ E. The plant was identified as *D. retrospiciens* in the Department of Botany and Microbiology, Cairo University, Egypt. The plant's voucher specimen (*Desmidorchis retrospiciens*: CU. Herb. Bot. 620) was placed at the herbarium.

In our study, fresh leaves of *D. retrospiciens* were washed with distilled water, then the leaves were air-dried at room temperature (25°C) for 4 days, avoiding direct sunlight exposure to reserve photosensitive phytochemicals. The aqueous leaf extract of *D. retrospiciens* was prepared using 30 g of fresh leaves. The leaves were washed, dried, cut, and crushed into powder. Then, 200 mL of water was added, and stirred at 50°C for 40 min. After that, the leaf extract was filtered using muslin cloth. The resulting solution was filtered using Whatman No. 1 filter paper to obtain a clear extract. The filtrate was stored at 4°C for further experiments (Supplementary Figure 1).

### Gas chromatography-mass spectrometry (GC–MS)

GC–MS analysis was carried out using an Agilent 7,890 gas chromatograph coupled to an Agilent 5,975 quadrupole mass detector equipped with a HP-5MS capillary column. The oven temperature was primarily kept at 45°C, and then increased to 175°C to 250°C. 1.0 μL of leaf extract was injected into the GC injection port.

### Biosynthesis and characterization of gold nanoparticles

1.0 mM of Hydrogen tetrachloroaurate (iii) trihydrate (HAuCl_4_.3H_2_O) was added to 30 mL of leaf extract. Then, the mixture was heated for 60 min until the color of the solution changed from yellow to ruby red. The biosynthesized AuNPs were separated from the reaction mixture via centrifugation. Finally, the AuNPs were washed, dried, and calcined at 200°C for 3 h (Supplementary Figure 1).

The biosynthesis of DR-AuNPs was primarily characterized using UV-visible (Shimadzu UV-1650, Shimadzu, Kyoto, Japan) spectrophotometer in a wavelength range of 200–800 nm. DR-AuNPs were further characterized using X'Pert PRO XRD and Fourier transform infrared (FTIR) spectroscopy (Perkin Elmer). To evaluate the stability of DR-AuNPs, zeta potential was measured. DR-AuNPs were suspended in sterile deionized water and zeta potential was measured using a Malvern Zetasizer Nano ZS system at a temperature of 25°C (Particle Technology Labs, Downers Grove, IL 60515, USA). The NPs were further examined using field-emission transmission electron microscopy (FE-TEM), energy-dispersive X-ray spectroscopy (EDX), and elemental mapping with a JEM-2100F (JEOL, Tokyo, Japan) instrument, at 200 kV.

### Microbiological studies

A methicillin-resistant *Staphylococcus aureus* (MRSA) ACLT 32571 was used in all microbiological experiments. These bacteria were selected because *S. aureus* infection is responsible for around 75% of clinical osteomyelitis. MRSA was cultured and maintained on specific media. For liquid culture medium, Mueller–Hinton Broth (MHB) was used to prepare bacterial suspensions, determine minimum inhibitory concentrations (MIC), minimum bactericidal concentrations (MBC), and conduct bacterial growth kinetics assays. For solid culture medium, Mueller–Hinton Agar (MHA) was used for disc diffusion assays and colony-forming unit (CFU) quantification. All media were sterilized by autoclaving at 121°C for 15 min.

Antibacterial activity of DR-AuNPs against MRSA was assessed by disc diffusion test according to the following method (Chandrasekaran et al., 2008). Briefly, 20 μL of DR-AuNPs at different concentrations (12.5, 25, and 50 μg/mL) was soaked on filter paper discs, dried, and placed in a culture plate containing MRSA, and incubated at 37°C for 24 h. The antibacterial effect was evaluated by measuring the diameter of the zone of inhibition. DMSO served as the negative control, while vancomycin (100 μg/mL) was used as the positive control.

### Minimum inhibitory concentration (MIC) assay

DR-AuNPs in successively decreasing concentrations (200 to 6.25 μg/mL) were added to microtitre-plates containing Mueller–Hinton broth following the method of Nkere and Iroegbu (2005). Optical densities were assessed at 600 nm using a microplate reader. DMSO was used as negative control, and vancomycin was used as positive control.

### Bacterial growth kinetics

Bacterial growth rate was detected using microtitre plates containing 10^5^ colony forming units (CFU/mL) of bacterial cells, loaded with DMSO, vancomycin (100 μg/mL), and DR-AUNPS (50 μg/mL). The optical density at 610 nm was recorded every 3 h intervals over 24 h (Sondi and Salopek-Sondi, 2004).

### Live/dead assay (SYTO9/PI staining)

After DR-AuNPs treatment, the MRSA cells were collected, stained with propidium iodide (PI, 25 μM) and SYTO9 (3.4 μM) for 20 min in the dark, and then examined using confocal microscopy (CLSM).

### Membrane potential

The modification of the membrane potential was examined using a membrane potential sensitive fluorochrome. After exposure to vancomycin and DR-AuNPs for 6 h, the MRSA cells were collected. The mixture of fluorescent probe DiBAC4 and bacterial suspension assessed using a fluorescence microplate reader (David et al., 2011).

### Reactive oxygen species (ROS) examination

ROS assessed by using the Nitroblue Tetrazolium (NBT) method as described (Becerra and Albesa, 2002). Briefly, 100 μL of MRSA (10^6^ CFU/mL) was mixed with Hanks' Balanced Salt Solution (HBSS) and treated with vancomycin or DR-AUNPS (MIC) for 3 h. Then, MRSA suspension was mixed with 500 μL of NBT and incubated for 25 min. One hundred microliter of hydrochloric acid was added to each suspension, and then it was centrifuged. The cell pellet mixed with HBSS and the color detected at 575 nm.

### Examination of leakage of ATP (adenosine triphosphate) and AKP (alkaline phosphatase)

AKP and ATP levels were assessed using the ATP and AKP test kits (Jiancheng Biology Engineering Institute, Nanjingjiancheng, China) following the manufacturer's instructions (Guo et al., 2021). Briefly, the *S. aureus* was cultured to the logarithmic growth stage (OD at 600 nm = 0.5), centrifuged, and washed in PBS. The MIC concentration of DR-AuNPs was mixed with MRSA and incubated in a shaking incubator at 37°C. A bacterial suspension without treatment was used as negative control group. Samples were collected at zero time and every 1 h. Subsequently, they were centrifuged at 3,000 rpm for 20 min. The supernatant was used to measure AKP contents using a kit. Furthermore, *S. aureus* cells were exposed to 8–64 μg/mL of DR-AuNPs for 1 h to observe the intracellular ATP levels.

### SEM and TEM

The alteration of bacterial morphology after treatment with DR-AuNPs was examined using a SEM Inspect F50 (ELECMI, Madrid, Spain) following the method described by Mendoza et al. (2017). TEM was performed as described using a JEM-1400Plus electron microscope (JEOL CANADA, INC), and pictures were collected employing a Veleta side-mounted camera (EMSIS ASIA Pte. Ltd, Singapore).

### Anti-biofilm activity of DR-AuNPs

#### Biofilm inhibitory concentration

A polystyrene microtiter plate comprising one mL of TSBS with DR-AuNPs (6.25–200 μg/mL) inoculated with MRSA (10^6^ CFU/mL) for 24 h. Then, planktonic cells were removed, and the biofilm cells were washed with a saline solution and stained with crystal violet solution. The absorbance was measured at 570 nm. The percentage of biofilm inhibition is determined by the following equation:


$$\begin{array}{l} \%\text{of inhibition = }[(\text{control }{\text{OD}}_{\text{570}}\text{ nm-treated }{\text{OD}}_{\text{570}}\text{ nm})\text{/}\\ \text{ control }{\text{OD}}_{\text{570}}\text{ nm}]\times \;\text{100}. \end{array}$$

The lowermost concentration of DR-AuNPs, which displayed maximum biofilm repression, was considered as the biofilm inhibitory concentration (BIC) (Valliammai et al., 2019).

#### Colony-forming unit and Alamar blue test

MRSA cells treated with DR-AuNPs (6.25–200 μg/mL) were diluted and distributed on a plate for CFU quantification. Tryptone Soya Agar (TSA) supplemented with 5% sucrose and Congo Red dye. The medium was prepared by adding 36 g/L of Tryptone Soya Agar, 50 g/L of sucrose, and 0.8 g/L of Congo Red dye. Afterwards, cells were suspended in Alamar blue solution, and fluorescence was detected at 530 and 590 nm for excitation and emission, respectively (Sarker et al., 2007).

#### Light microscopic examination

For light microscopic investigation, MRSA biofilm was developed on glass/stainless steel slides submerged in TSBS in the presence of DR-AuNPs. Afterward, the slides were washed with saline and stained with crystal violet for 30 min. They were observed under a light microscope at ×400 magnification.

#### SEM of bacterial biofilm

Diluted human blood Plasma was used. Titanium slides were coated with a plasma solution and incubated at 4°C. Then, the plasma solution was removed, and biofilm was developed on the titanium surface in the presence of DR-AuNPs. Then, the slides washed, fixed with 2.5% glutaraldehyde, dehydrated with ascending concentrations of ethanol, and dried. Finally, the slides were exposed to gold sputtering and examined as described by Walker and Horswill (2012).

#### Congo red agar assess

Tryptone Soya Agar (TSA) supplemented with 5% sucrose and Congo Red dye. The medium was prepared by adding 36 g/L of Tryptone Soya Agar, 50 g/L of sucrose, and 0.8 g/L of Congo Red dye. Then, DR-AuNPs or DMSO were mixed into the media and added to Petri plates. The bacterial culture was inoculated on the plates and incubated for 24 h. Finally, the plates were examined as described (Knobloch et al., 2002).

#### Examination of biofilm development in flow conditions

The BioFlux 1000 system was employed to examine the active biofilm development under flow conditions as described (Passos et al., 2021). Primary, the microfluidic channels of the plate were primed with TSBG medium from the inlet wells. The log-phase MRSA cells (10^8^ CFU/mL) were flown into the channels. After the initial attachment of MRSA, DR-AuNPs and vancomycin (MIC) were added. MRSA biofilm formation was automatically monitored for 15 h with BioFlux Montage software 2.3.

#### Examination of three-dimensional structure of MRSA biofilms using CLSM

MRSA was inoculated into a glass-bottomed dish containing 2 mL of TBSG. The unattached cells were removed, and the developed biofilms were washed with saline and stained with SYTO9 and propidium iodide. A TCS SP8 microscope was used to obtain fluorescent pictures, evaluate biofilm thickness, and examine fluorescence intensities.

### Cell cultures and cell viability assay

Mouse bone marrow mesenchymal stem cells (mBMSCs) were isolated from long bones of mice as described (Abdallah et al., 2019). Cells were cultured in RPMI-1640 medium supplemented with 1% penicillin/streptomycin (P/S) and 10% fetal bovine serum (FCS) (Thermo Fisher Scientific GmbH). Cell viability of BMSCs in the absence and presence of DR-AuNPs was measured using MTT cell proliferation assay kit (Sigma-Aldrich) according to the manufacturer's instructions.

### Osteoblast differentiation

BMSCs were induced to differentiate into osteoblasts using α-minimum essential medium (α-MEM; Thermo Fisher Scientific GmbH) supplemented with 10 mM β-glycerol-phosphate, 50 mg/mL of vitamin C (Sigma-Aldrich), 10% FBS, and 1% penicillin/streptomycin (P/S). Osteogenic induction was maintained for 12 days, with the medium being changed every 2 days.

### Alkaline phosphatase (ALP) activity assay and staining

BMSCs were induced to undergo osteoblast differentiation in 96 96-well plates with or without DR-AuNPs (100 μg/mL), for 1 week. ALP activity was measured as described (Abdallah et al., 2019). CellTiter-Blue^®^ cell viability assay was used to determine cell viability according to the manufacturer's instructions. The value of ALP activity was represented after normalization to the cell viability. For ALP staining, BMSCs were fixed with acetone/citrate buffer (pH 4.2) and stained with Napthol-AS-TR-phosphate solution (Sigma-Aldrich) for 1 h at room temperature (Abdallah and Ali, 2020).

### Alizarin red S staining and quantification

BMSCs were induced to undergo osteoblast differentiation for 12 days, fixed with 70% ice-cold ethanol for 1 h at −20°C, and stained with Alizarin red (ALZ) for 10 min at room temperature (RT). For calcium deposition quantification, ALZ was eluted with 10% cetylpyridinium chloride for 1 h at RT, and the absorbance was measured at 570 nm. The data are presented after normalization to cell number.

### *In vivo* bone-formation assay

Mouse BMSCs (5 × 10^5^), either without (control) or with DR-AuNPs (100 μg/mL), were mixed with 40 mg of hydroxyapatite/tricalcium phosphate powder (HA/TCP) with 100 μL of standard growth medium. Mixture of cells with HA/TCP was implanted subcutaneously into the dorsal surface of 2-month-old female immuno-deficient NOD/SCID mice under anesthesia, induced by an intraperitoneal injection of ketamine/xylazine (100/10 mg/kg). Each mouse received four or six implants per treatment. Mice were sacrificed after 8 weeks of transplantation, and the implants were dissected out. Implants were fixed in 4% formaldehyde, decalcified in formic acid for 3 days, paraffin-embedded, and sectioned into 4-μm sections. Sections were stained by eosin/hematoxylin as described previously (Abdallah et al., 2008). The percentage of total bone formation per total implant was measured using the visual scoring method as described (Kaigler et al., 2006).

### RNA extraction and quantitative real-time PCR (qPCR)

Total RNA was extracted using the TRIzol single-step method (Thermo Fisher Scientific). cDNA was synthesized from 2 μg of total RNA using the revertAid H minus first strand cDNA synthesis kit according to the manufacturer's instructions (Fermentas, St Leon-Rot, Germany). Gene expression analysis performed by using Applied Biosystems 7500 Real-Time system with Fast SYBR^®^ Green Master Mix (Applied Biosystems, California, USA). β*-Actin* and *Hprt* mRNA expression used as reference genes. Gene expression measured by using a comparative CT method [(1/(2delta-CT) formula, where delta-CT is the difference between CT-target and CT-reference] with Microsoft Excel 2007^®^ (Abdallah and Ali, 2019).

### Statistical analysis

Values of at least three independent experiments expressed as mean ± SD (standard deviation). Power calculation used for 2-samples with unpaired Student's *t*-test (2-tailed) assuming equal variation in the two groups. ^*^P < 0.05, and ^**^P < 0.005 were considered statistically significant.

## Results

### Identification of phytochemical components of *D. retrospiciens*

**Figure 1:**
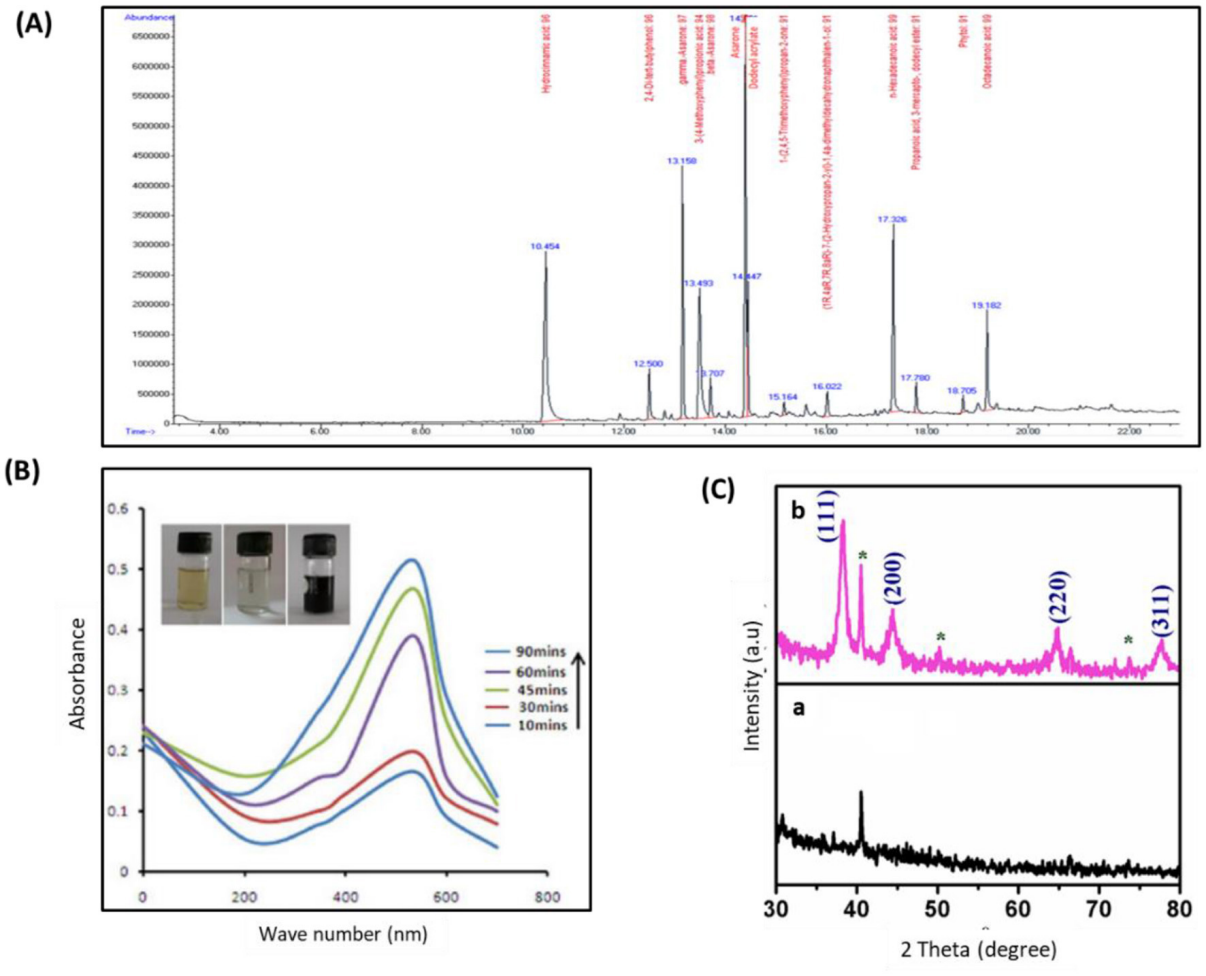
Characterization of phyto-fabricated DR-AuNPs using GC–MS, UV-vis spectral, and XRD analysis. **(A)** GC–MS chromatogram of aqueous extract of *D. retrospiciens*. **(B)** UV-vis spectral examination and color intensity of DR-AuNPs at different time intervals of 45 min. The inset displays the color alteration from ruby red to a dark black color. **(C)** XRD patterns of (a) *D. retrospiciens* aqueous leaf extract and (b) DR-AuNPs biosynthesized from *D. retrospiciens* (*bioinorganic components occur in the leaf extract).

GC–MS examination of the leaf extract of *D. retrospiciens* (Figure 1A) presented 13 peaks, which designated the occurrence of 13 phytochemical components. On assessment of the mass spectra of the components with the NIST library, the most predominant compounds were hydrocinnamic acid (peak value 15.43%) and asarone (peak value 14.40%); afterward, 3-(4-methoxyphenyl) propionic acid (peak value 13.49%) and gamma-asarone (13.15%) (Table 1).

**Table 1:** Phytochemicals in the GC–MS-analyzed aqueous leaf extract of *Desmidorchis retrospiciens*.

**No**.	**Compound name**	**Retention time (min.)**	**Area (%)**
1	Hydrocinnamic acid	10.45	15.43
2	2,4-Di-tert-butylpheno	12.50	1.22
3	gamma-Asarone	13.15	8.83
4	3-(4-Methoxyphenyl)propionic acid	13.49	9.53
5	beta-Asarone	13.70	1.12
6	Asarone	14.40	15.33
7	Dodecyl acrylate	14.44	4.22
8	1-(2,4,5-Trimethoxyphenyl)propan-2-one	15.16	0.65
9	(1R,4aR,8aR)-7-(2-Hydroxypropan2-yl)-1, dimethyldecahydronaphthalen-1-ol	16.02	0.98
10	n-Hexadecanoic acid	17.32	8.23
11	Oxalic acid, propyl tetradecyl ester	17.78	0.97
12	Phytol	18.70	0.45
13	Octadecanoic acid	19.17	3.38

### Biosynthesis mechanism of DR-AuNPs

To phyto-fabricate DR-AuNPs, the plant extract of *D. retrospiciens* was employed as a reducing, stabilizing, and capping agent. The development of DR-AuNPs was followed by the detection of color alteration. The reaction mixture's color change from yellow to ruby red designated the development of AuNPs. This color change happened as a result of the surface Plasmon resonance action (Supplementary Figure 1).

### Characterization of phyto-fabricated DR-AuNPs

**Figure 2:**
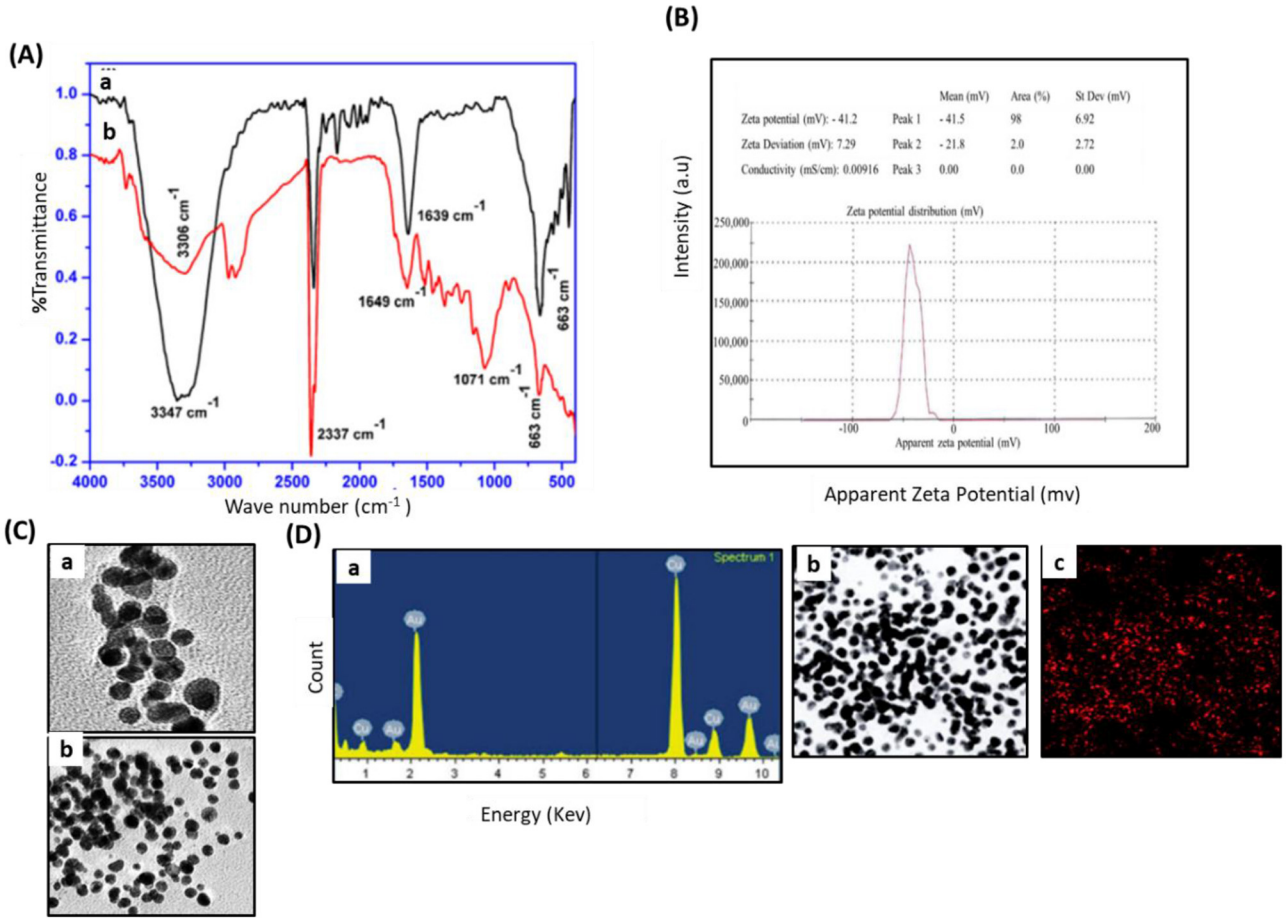
Characterization of phyto-fabricated DR-AuNPs using FTIR, Zeta potential, TEM, and EDX spectrum analysis. **(A)** FTIR spectra for (a) aqueous leaf extract of *D. retrospiciens* and (b) phyto-engineered DR-AgNPs. **(B)** Zeta potential distribution of DR-AuNPs biosynthesized by aqueous leaf extract of *D. retrospiciens*. **(C)** TEM pictures of DR-AuNPs at 10 nm (a), at 20 nm (b). **(D)** EDX spectrum of DR-AuNPs (a), elemental mapping: electron micrograph area of DR-AgNPs (b), spreading of Au element (c).

The UV-visible spectral examination established the biosynthesis and stability of the DR-AuNPs. At a 534 nm wavelength, the optimum DR-AuNPs biosynthesis was investigated at 60°C, pH 7 with 1 mM HAuCl4, and 45 min incubation period (Figure 1B). The reduction of AuCl4^−^ was evident from the color alteration, and the biosynthesis process completed in 90 min, with a ruby-red color indicating the development of DR-AuNPs. The XRD of the leaf extract and DR-AuNPs are displayed in Figure 1C, where no matching peaks are observed for gold metal in the plant extract. However, Figure 1C, b display several Bragg reflection peaks at 38°, 44°, 64°, and 77°, which are indexed by planes (111), (200), (220), and (311), respectively, corresponding to the face-centered cubic orientation of Au. The (111) plane of DR-AuNPs is detected more strongly than other planes. So, it is employed to estimate the particle size of Scherrer's equation as 19 nm. Figure 2A displays the FTIR spectra for aqueous leaf extract of *D. retrospiciens* and DR-AuNPs. The spectra of plant extract display strong peaks at 3,346 cm^−1^ (O-H stretch), 2,337 cm^−1^ (C = N stretch), 1,629 cm^−1^ (C = C stretch), 1,070 cm^−1^ (C-N stretch), and 665 cm^−1^ (C-S stretch). These peaks authorize several constituents of *D. retrospiciens* leaf extract. The spectra of DR-AuNPs also display all these strong peaks with shift in O-H stretching from 3,346 to 3,305 cm^−1^ and C-C stretching from 1,629 to 1,647 cm^−1^. These shifts could be due to the reduction of gold ions to gold-capped phyto-fabricated DR-AuNPs. Zeta potential examination can evaluate the surface charges of NPs and assess the repulsion between the charged particles in colloid state. Peak 1 has an average of −41.3 ± 6.90 mV, and its area represented 98% of the potential distributions. The zeta potential of peak 2 was 21.7 ± 2.70 mV; though, it is only responsible for 2% of the potential distributions. The zeta potential of all the examined NPs was 41.1 ± 7.27 mV (Figure 2B). These results indicated that DR-AuNPs were stable. The FE-TEM pictures presented a perfect morphology of phyto-fabricated DR-AuNPs. The size of DR-AuNPs ranged from 10 to 20 nm with spherical shape (Figure 2C, a and b). The EDX examination of DR-AuNPs presented an optical absorption peak at 2.2 keV (Figure 2D, a), which is the characteristic absorption of metallic AuNPs. Elemental mapping investigation of DR-AuNPs presented great dispersal of Au in the SEM picture of AuNPs pellet solution (Figure 2D, b and c).

### Antibacterial action of DR-AuNPs

**Figure 3:**
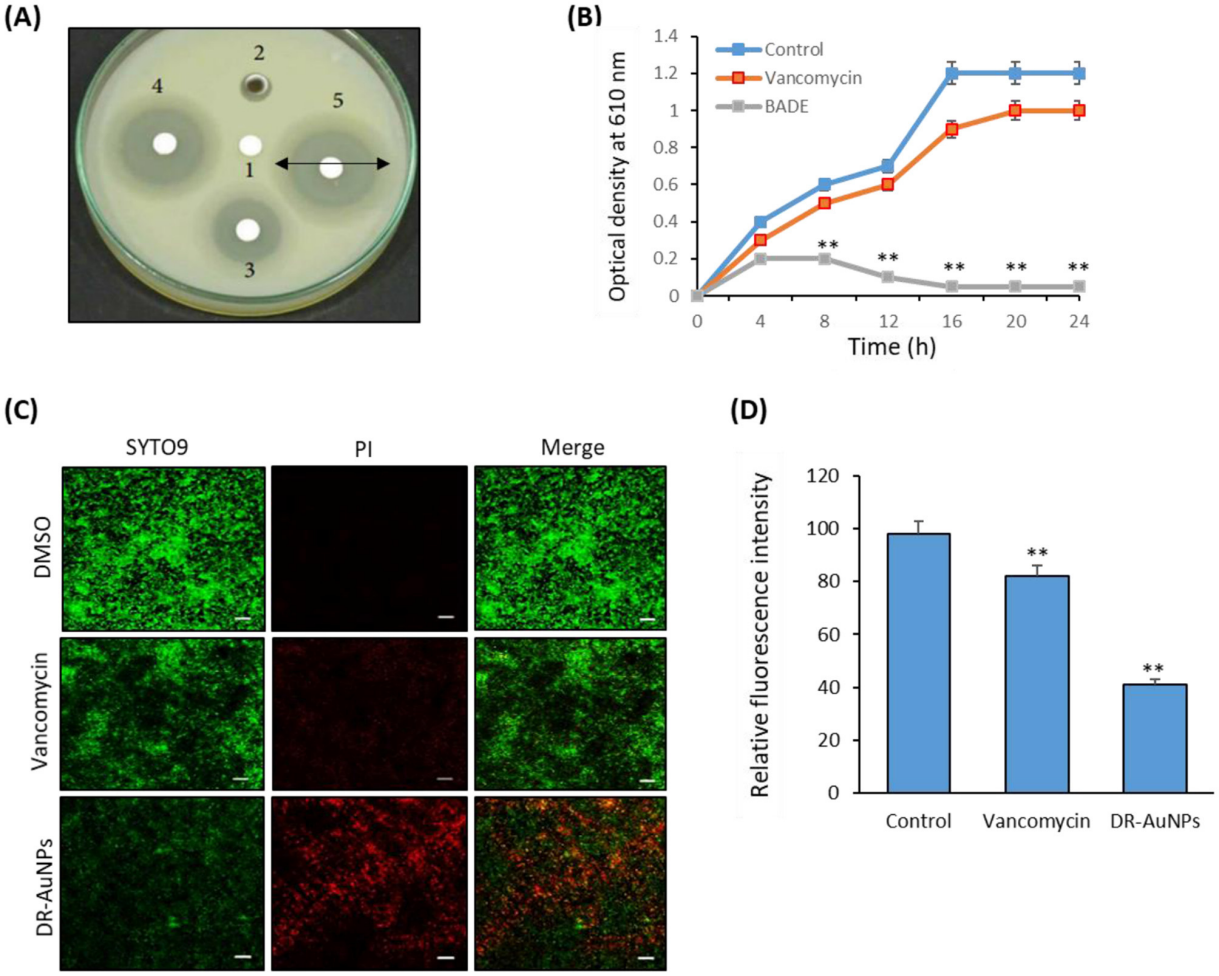
Antibacterial activity of DR-AuNPs against MRSA. **(A)** Disc diffusion assay demonstrating the antibacterial action showing zone of antibacterial inhibition against MRSA by DMSO (negative control) (1), DR-AuNPs (12.5 μg/ml) (2), Vancomycin (positive control) (3), DR-AuNPs (25 μg/ml) (4), and DR-AuNPs (50 μg/ml) (5). Data are represented as the mean zone of inhibition in mm. **(B)** The bactericidal activity of DR-AuNPs (50 μg/ml) and vancomycin (100 μg/ml) against MRSA. Data are the means ± SD (*n* = 3). **(C)** Live/dead (SYTO9/PI) stain pictures of MRSA treated with DR-AuNPs (50 μg/ml) and vancomycin (100 μg/ml) for 6 h using confocal microscopy. Red, dead bacteria. Green signal, living bacteria. PI, propidium iodide. Scale bar = 20 μm. **(D)** The membrane potential of MRSA exposed to DR-AuNPs (50 μg/ml) and vancomycin (100 μg/ml) for 6 h was assessed by membrane potential sensitive fluorochrome DiBAC4 (mean ± SD, *n* = 3). **p < 0.005.

The minimum inhibitory concentration (MIC) of DR-AuNPs against MRSA was determined using a microdilution assay in Mueller–Hinton broth. Our results show that DR-AuNPs inhibited bacterial growth at concentrations ≥50 μg/mL, indicating that 50 μg/mL is the MIC. A dose-dependent increase in the size of the inhibition zone was observed from 12.5 μg/mL, with the greatest size achieved at 50 μg/mL. However, the inhibition zone was not detected in control loaded with DMSO, while vancomycin displayed higher growth suppression (Figure 3A). Additionally, the growth of MRSA treated with DR-AuNPs decreased after 2 h of incubation as compared with the control (Figure 3B). DR-AuNPs at a concentration of 50 μg/mL completely suppressed MRSA. Moreover, the antibacterial activity of DR-AuNPs was confirmed by a Live/Dead fluorescent staining test, where the number of dead MRSA (red) significantly increased after exposure to DR-AuNPs and vancomycin by 82% and 41%, respectively, as compared with control (Figures 3C, D). The aqueous leaf extract of *D. retrospiciens* (without gold salt) was tested as a control in MIC inhibition assay. It did not exhibit significant antibacterial action against MRSA at the tested concentrations. These results support that the observed effects are primarily due to DR-AuNPs (Data not shown).

### Effect of DR-AuNPs on MRSA ultrastructure modification

**Figure 4:**
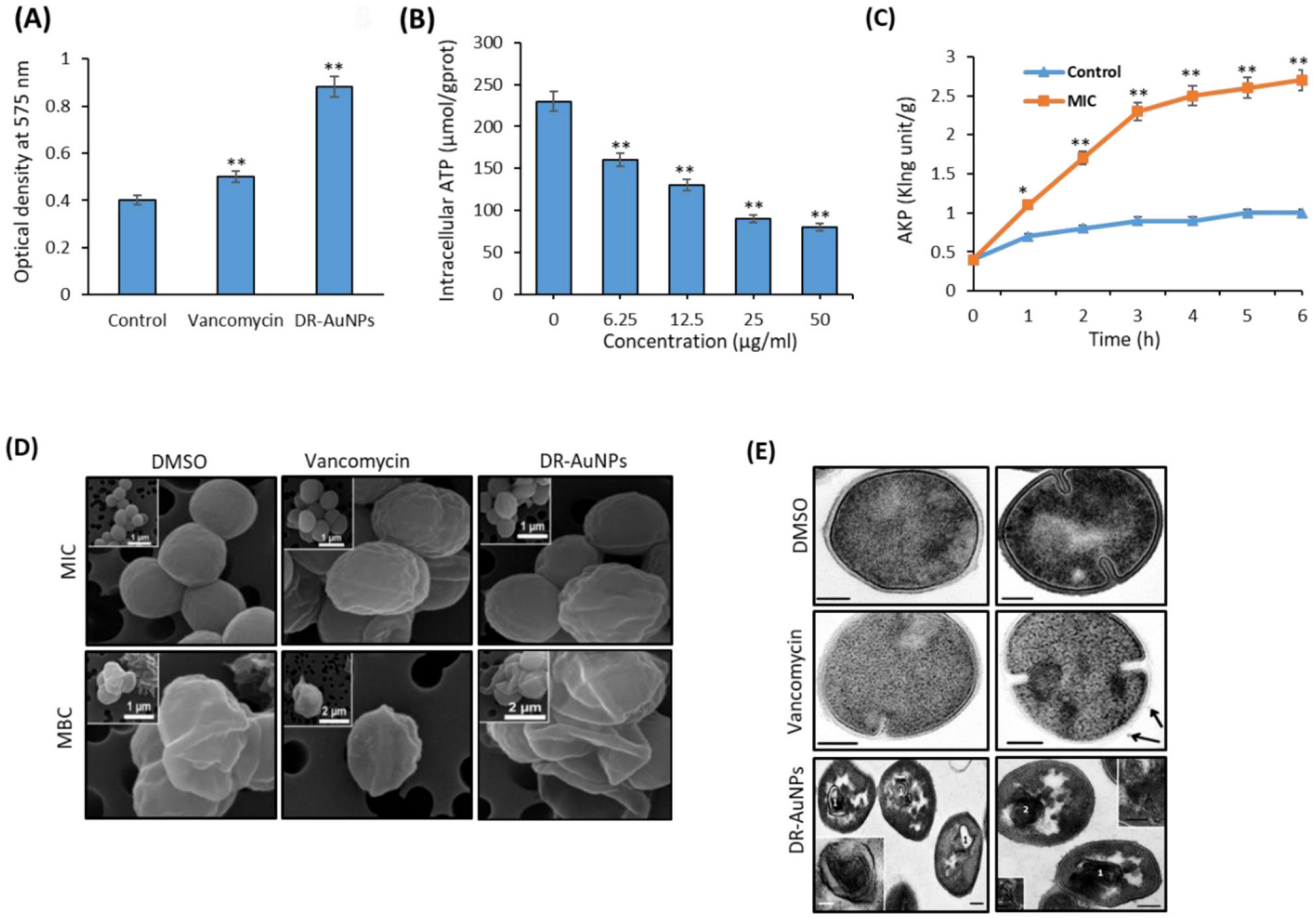
Effect of DR-AuNPs on bacterial ROS production. **(A)** The intracellular ROS levels of the DR-AuNPs and vancomycin-treated MRSA. Data are the means ± SD (*n* = 3). **(B)** ATP release in MRSA treated with DR-AuNPs. **(C)** AkP release in MRSA treated with DR-AuNPs. Values are expressed as mean ± SD (*n* = 3), **P < 0.005 compared to control. **(D)** Scanning electron microscopy pictures of MRSA cells treated for 24 h with DMSO (control sample) and MIC or MBC of DR-AuNPs and vancomycin. **(E)** Ultrastructure of MRSA cells incubated for 30 min with DMSO (negative control). Scale bars =100 nm, vancomycin (positive control, 100 μg/ml). Arrow displays MV. Scale bars = 200 nm, and DR-AuNPs (50 μg/ml). (1) Multi-membrane structures. The insert displays these structures at higher magnification. (2) “Decorated” fibers, inserts on image displays “decorated” fibers at higher magnification. Scale bars = 200 nm. Values are mean ± SD of three independent experiments, **P < 0.005, compared to control. *p < 0.005

We studied the effect of DR-AuNPs on bacterial ROS production. Treatment of MRSA with DR-AuNPs resulted in a greater (66%) intracellular ROS accumulation compared to vancomycin treatment (30%), and was significantly higher than the control (Figure 4A).

We evaluated the differences in ATP levels by MRSA after exposure to various concentrations of DR-AuNPs (6.25–50 μg/mL). DR-AuNPs suppress ATP in MRSA in a dose-dependent manner (Figure 4B). AKP is used as a crucial sign for measuring the integrity of the cell wall. As presented in Figure 4C, DR-AuNPs (50 μg/mL) expressively increase the discharge of AKP after 1 h, as compared to control. Moreover, the discharge of AKP rose to 2.7 King units/g after 6 h of exposure (Figure 4C). Remarkably, DR-AuNPs had a major influence on both ATP and AKP in MRSA compared to vancomycin.

MRSA cells treated with DMSO displayed a spherical form and a preserved cell membrane. Nevertheless, after treatments with DR-AuNPs and vancomycin at their respective MICs for 24 h, the morphology of MRSA was altered as assessed by SEM (Figure 4D). Part of the cell's peptidoglycan structure appeared to be depressed, representing an early destruction. MRSA cells treated with MBC concentrations appeared distorted and crumpled, demonstrating that the intracellular components had been released. There was a reduced number of MRSA cells. It was difficult to find the ones treated with DR-AuNPs, possibly due to the significant destruction of the MRSA peptidoglycan layer and cell membrane. Consequently, cell death and dispassion from the filter holder (Figure 4D). The decrease in cell size and diameter detected could be due to the leak of cytosolic fluids outside the cells. The TEM investigation confirms the action of DR-AuNPs on the morphology of MRSA cells. The ultrastructure of MRSA cells treated with DMSO did not markedly differ from the published data. MRSA cells in ultrathin sections had a shape close to spherical and a diameter of 600–900 nm. Approximately half of the cells displayed the development of septa, indicating cell division (Figure 4E). However, MRSA cells treated with vancomycin were characterized by the development of multi-membrane structures that did not look like mesosomes. These structures are comprised of thin membranes and are found in areas of septum establishment and on ultrathin sections, resembling a loose accumulation of unordered membranes that occasionally comprise electron-dense material (Figure 4E). Interestingly, exposure of MRSA cells to DR-AuNPs resulted in the formation of multilayered membrane structures that differ in form and size. All these structures could be classified into two forms: membranes that were firmly fixed together and had a high electron density; membranes had a normal electron density, and the space between them was filled with a homogeneous substance of normal electron density that looked like the nearby cytoplasm (Figure 4E).

### Anti-biofilm activity of DR-AuNPs

**Figure 5:**
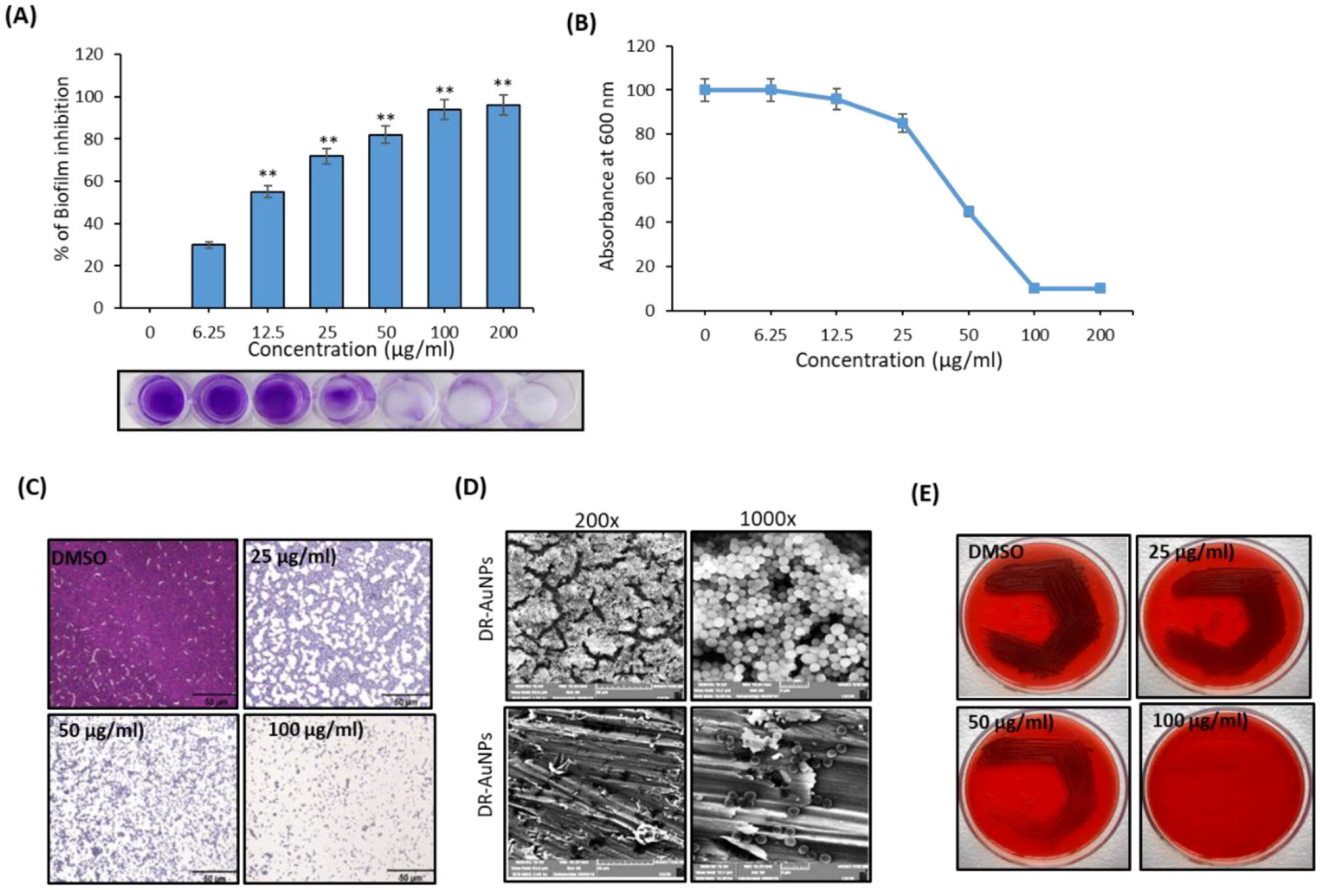
Anti-biofilm activity of DR-AuNPs against MRSA. **(A)** Effect of DR-AuNPs on the biofilm development of MRSA as examined by crystal violet staining represented as a percentage of biofilm inhibition. The lowermost pictures display the corresponding crystal violet-stained MRSA biofilm. The error bars indicate standard deviations. The asterisks represent statistical significance (*P* < 0.05). **(B)** The growth of MRSA OD 600 nm. The error bars indicate standard deviations. The asterisks represent statistical significance (*P* < 0.05). **(C)** Light microscopic pictures (400×) showing the dose-dependent anti-biofilm activity of DR-AuNPs against MRSA. Scale bar = 50 μm. **(D)** Scanning electron microscopy pictures displaying the decrease of adherence of MRSA on plasma-coated titanium surface after treatment with DR-AuNPs (100 μg/ml) at two magnifications. **(E)** Congo red test demonstrating the decrease in the synthesis of polysaccharide intracellular adhesion after treatment with different concentrations of DR-AuNPs. Values are mean ± SD of three independent experiments, **P < 0.005, compared to control.

DR-AuNPs exhibited a dose-dependent anti-biofilm effect against MRSA, achieving a biofilm suppression of 92% at a concentration of 100 μg/ml, as measured by the crystal violet assay. After this concentration, the biofilm suppression was not significantly increased (Figures 5A, B).

The anti-biofilm activity of DR-AuNPs was additionally evaluated by microscopic examination. Light micrographs revealed a continued decrease in surface coverage with rising concentrations of DR-AuNPs (Figure 5C). In addition, the development of MRSA biofilm in the presence of DR-AuNPs was examined using SEM. The control surface was detected to be enclosed by MRSA biofilm, while a monolayer of detached MRSA cells was detected in the DR-AuNPs-exposed surface (Figure 5D). Moreover, the Congo red agar test revealed that the bacterial cells seemed black in the absence of DR-AuNPs. However, DR-AuNPs treatment progressively repressed the black coloration in a concentration-dependent manner (Figure 5E).

### Effect of DR-AuNPs on MRSA biofilm development under flow conditions

**Figure 6:**
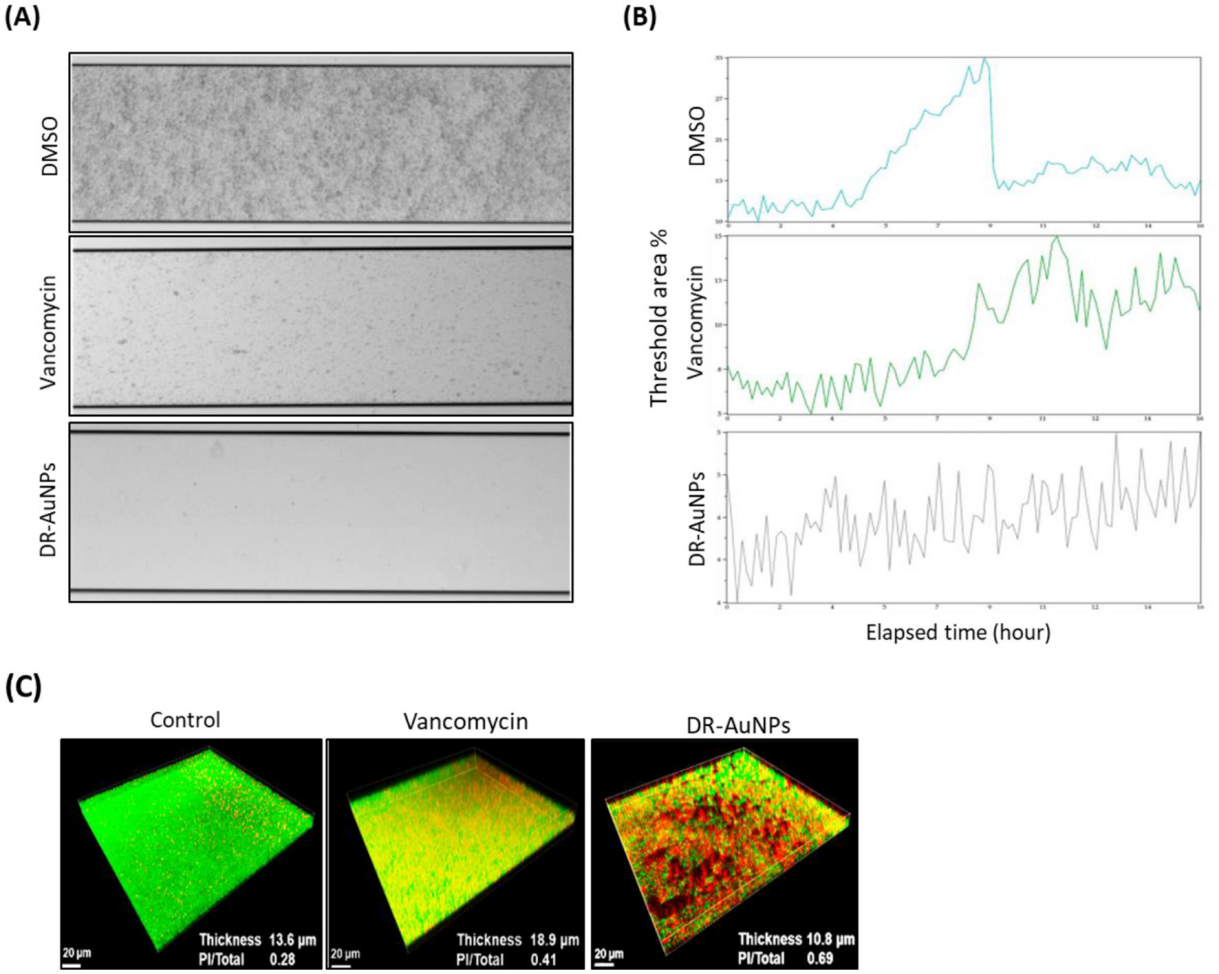
Effect of DR-AuNPs on MRSA biofilms. **(A)** Inhibition of biofilm establishment of the MRSA by DR-AuNPs under a flowing condition (BioFlux 1000 system). The 24-h biofilm developed in the channels was snapped. **(B)** The amount of biomass was examined in real-time using BioFlux Montage software 2.3. **(C)** Live/dead staining. Fifty μg/mL of DR-AuNPs, 100 μg/mL of vancomycin, or 0.1% DMSO (negative control) were added to a pre-formed 24 h biofilm of MRSA in fluoroDishes. After 24 h incubation, the biofilm thickness and fluorescence intensities were assessed.

The effect of DR-AuNPs on active biofilm formation was investigated employing a BioFlux 1000 system. In the DMSO group, MRSA cells adhered firmly to the bottom of the channel (Figure 6A) and the biomass of the MRSA biofilm progressively increased, reaching a peak at 8.3 h (Figure 6B), with ~32% threshold areas. However, in the channels exposed to vancomycin, biofilm biomass displayed a moderate area. Interestingly, in the DR-AuNPs group, a significant suppression of biofilm development (3% threshold area) was observed and no clear peak was detected (Figures 6A, B).

### Effect of DR-AuNPs on MRSA mature biofilm and cell viability

CLSM indicated that the mature biofilm of MRSA following exposure to DMSO had an average thickness of 13.3 μm and retained its complete structure. Exposure to vancomycin (MIC) led to an increase in biofilm thickness to 18.9 μm. Furthermore, the quantity of dead MRSA cells in the DMSO and vancomycin groups was about 0.25 and 0.40, respectively. However, it increased to ~0.70 in the DR-AuNPs-treated mature biofilm (Figure 6C). Interestingly, the exposure to DR-AuNPs (MIC) resulted in a significant decrease in average thickness (10.5 μm) and a ruined structure (Figure 6C).

### DR-AuNPs stimulate the differentiation of mBMSCs into osteoblast lineage

**Figure 7:**
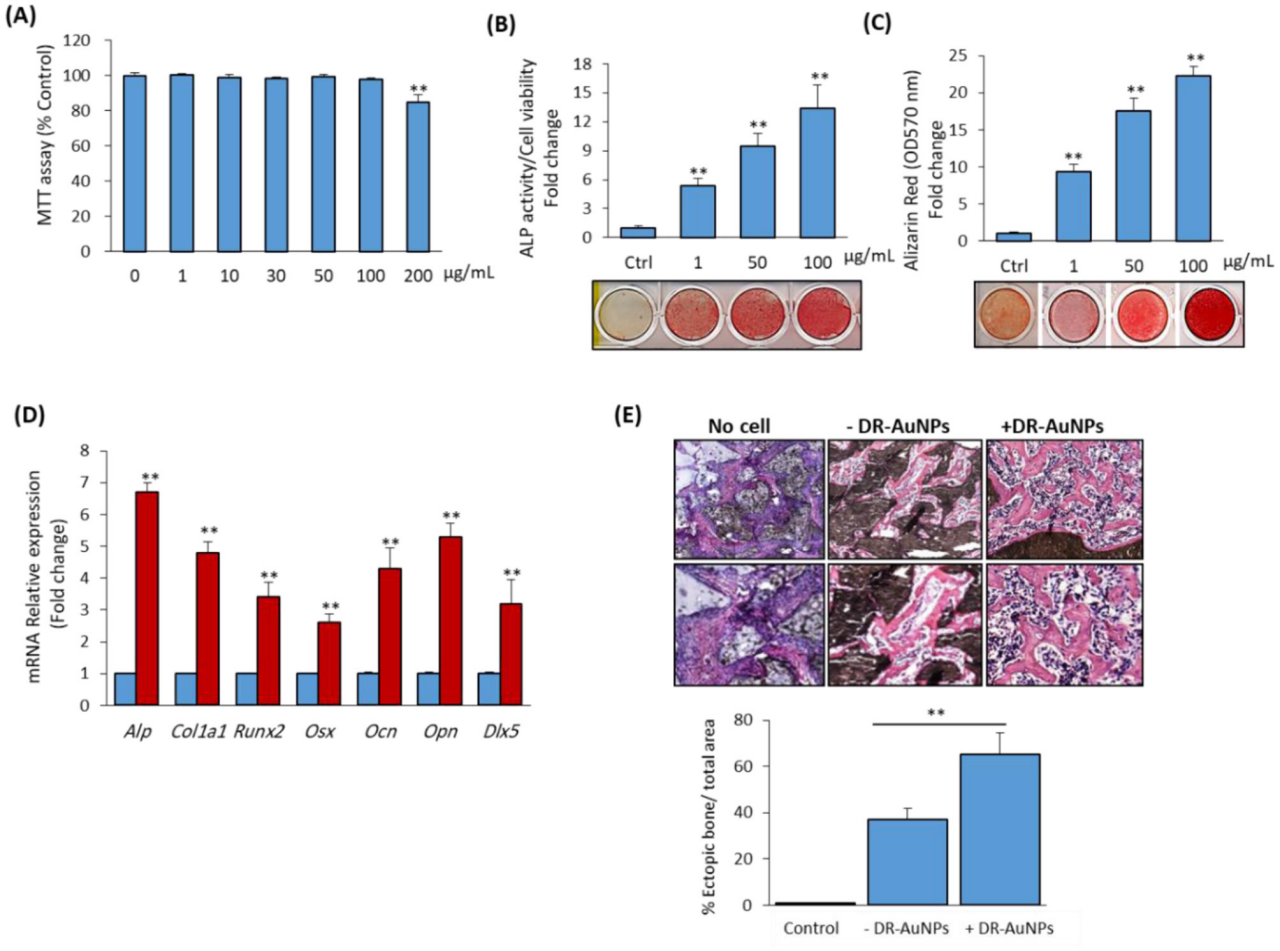
Stimulatory effect of DR-AuNPs on osteoblast differentiation of mBMSCs *in vitro* and *in vivo*. **(A)** Cytotoxicity of DR-AuNPs on primary mBMSCs. The effect of different concentrations of DR-AuNPs on cell viability was assessed using the MTT assay after 48 h of treatment. Dose-dependent stimulatory effect of DR-AuNPs on **(B)** ALP activity and **(C)** matrix mineralization of mBMSCs. Cells were induced to osteoblast differentiation in the absence (Ctrl) or presence of different concentrations of DR-AuNPs for 12 days. ALP and Alizarin red staining images are shown. **(D)** qPCR analysis of the expression of osteoblast marker gene expression in mBMSCs after 12 days of osteogenic induction without or with DR-AuNPs (100 μg/mL). Gene expression was normalized to reference genes and represented as fold change compared to induced cells without DR-AuNPs **(E)**. Histological analysis of *in vivo* ectopic bone formation by mBMSCs loaded on HA/TCP and treated without or with DR-AuNPs (100 μg/mL) after 8 weeks of subcutaneous transplantation in immunodeficient mice. Implants without cells were used as negative control. The graph shows the quantification of new bone formation as a percentage of the total area. Values represent the mean ± SD of three independent experiments, **P < 0.005, compared to control.

To study the osteo-inductive effect of DR-AuNPS, we first examined its cytotoxicity on mouse bone marrow-derived mBMSCs. As shown in Figure 7A, DR-AuNPS did not display any cytotoxicity on mBMSCs viability up to a concentration of 100 μg/mL (Figure 7A), as assessed by MTT assay. DR-AuNPS significantly reduced cell viability at a concentration of 200 μg/mL. DR-AuNPS displayed strong *in vitro* osteoinductive effect as revealed by its stimulatory effect on increasing ALP activity (Figure 7B) and matrix mineralization production (Figure 7C) by mBMSC. In addition, DR-AUNPS upregulated the mRNA expression of early and late bone marker genes as measured by qPCR analysis (Figure 7D). We further examined the effect of DR-AuNPS on enhancing the ectopic bone formation capacity of mBMSCs *in vivo*. Interestingly, as shown in Figure 7E, treatment of mBMSCs with DR-AuNPS was found to increase their *in vivo* bone formation capacity in a dose-dependent manner.

### DR-AuNPs exerts osteoinductive activity via stimulating ERK1/2 signaling pathway

**Figure 8:**
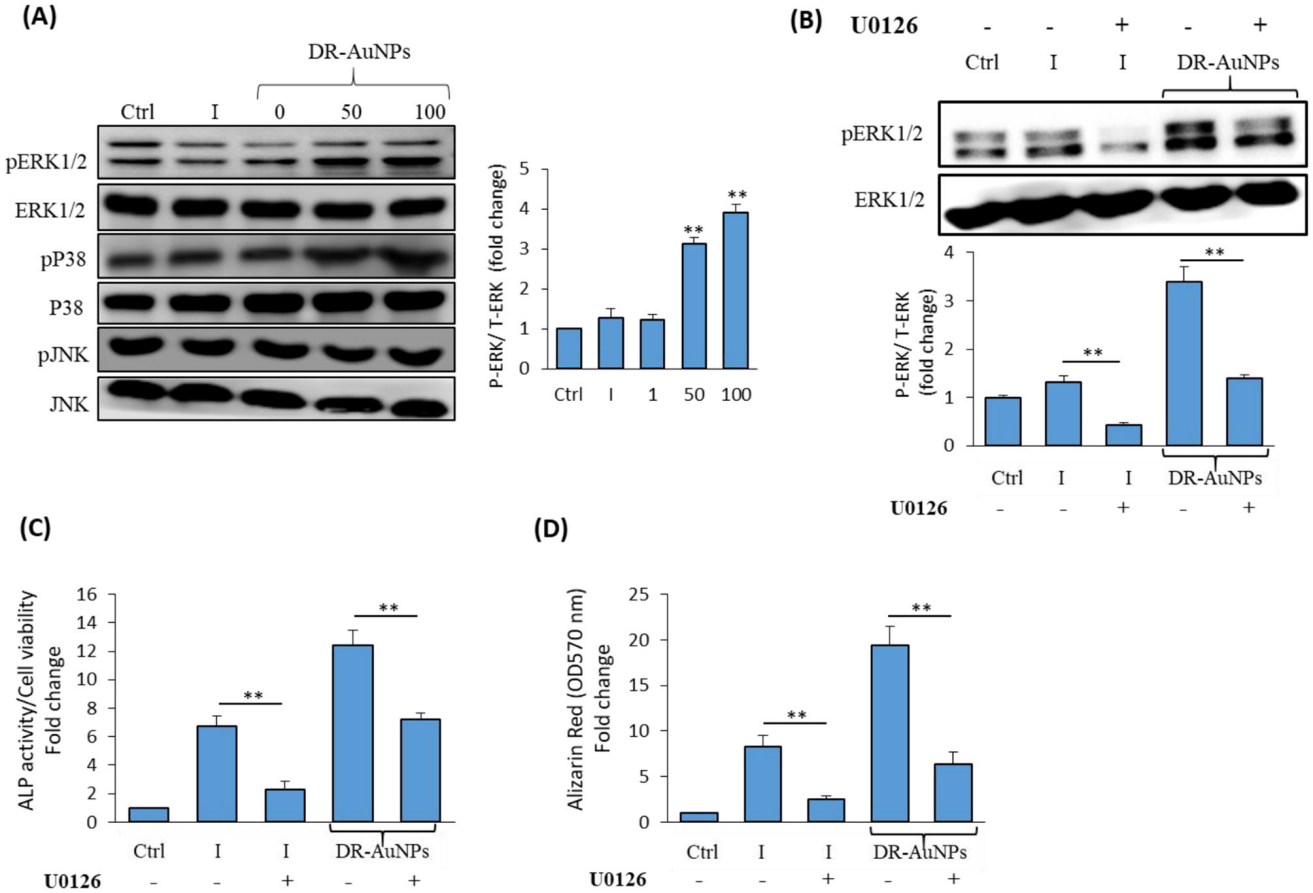
The osteoinductive effect of DR-AuNPs is mediated by MAPK/ERK signaling pathway. **(A)** Effect of DR-AuNPs on MAPK-related proteins expression in mBMSCs as measured by Western blot analysis. Cells were induced to osteoblast differentiation in the absence (I) or the presence of different concentrations of DR-AuNPs for 4 h. The graph illustrating the quantification of ERK1/2 band density is shown. **(B)** Western blot analysis of the inhibitory effect of the specific ERK1/2 inhibitor, U0126, on DR-AuNPs-induced ERK1/2 phosphorylation during osteogenesis in mBMSCs. Cells pre-treated with U0126 (10 μM) and induced with an osteogenic cocktail for 4 h, without (I) or with DR-AuNPs (100 μg/mL). **(C)** Blocking of ERK1/2 activation by U0126 in mBMSC significantly suppressed the stimulatory effect of DR-AuNPs on osteogenesis as assessed by the quantification of ALP activity and **(D)** matrix mineralization. Values represent mean ± SD of three independent experiments, **P < 0.005, compared to control non-induced cells.

We investigated the signaling pathway that regulates the stimulatory effect of DR-AuNPs on osteogenesis. As shown in Figure 8A, DR-AuNPs significantly stimulate the activation of ERK1/2 phosphorylation in a dose-dependent manner during osteoblast differentiation of mBMSCs. On the other hand, the DR-AuNPs did not affect the phosphorylation of other members of the MAPK family, including p38 or JNK proteins, as assessed by Western blot analysis (Figure 8A). The stimulatory effect of DR-AuNPs on ERK1/2 activation was further confirmed by using an ERK1/2 specific inhibitor, U0126 (Figure 8B). Interestingly, blocking of ERK1/2 activation by U0126 during osteoblast differentiation of mBMSCs showed that inhibited DR-AuNPs-induced ALP activity and matrix mineralization by 42% and 63%, respectively (Figures 8C, D). Thus, the osteo-inductive effect of DR-AuNPs is mediated via activating ERK1/2 signaling.

## Discussion

Osteomyelitis is challenging to diagnose and treat due to its biological complexities and the heterogeneity of infectious presentations, as well as its pathophysiology. In this research, we phyto-fabricated AuNPs using extract of *D. retrospiciens* and established their antibacterial and osteo-inductive activities as a promising bioactive agent for osteomyelitis treatment. Our data provide a novel and promising strategy for treating osteomyelitis using green AuNPs as compared with reported complicated compositions of nanoparticles.

We phyto-fabricated DR-AuNPs for the first time using aqueous leaf extract of *D. retrospiciens*. Interestingly, *D. retrospiciens* presented medicinal properties and is known for its antibacterial, antiviral, and wound healing activities (Al-Robai et al., 2022). The GC-MS analysis of *D. retrospiciens* extract revealed the presence of 13 major bioactive phytochemicals with pharmacological activities, including antimicrobial properties. For example, hydroxycinnamic acid, n-hexadecanoic acid, and 2,4-di-tert-butylphenol displayed antioxidant, anti-inflammatory, and antimicrobial actions (Taofiq et al., 2017; Zhao et al., 2020; Aparna et al., 2012). In addition, asarone presents a wide range of pharmacological activities, including antioxidant, anti-inflammatory, antidiabetic, antifungal, anti-ulcer, anti-allergic, and wound healing activities (Lam et al., 2017). Thus, these phytochemicals could act as green bio-reducing and capping agents for biosynthesis of DR-AuNPs.

DR-AuNPs was established by a strong peak at 534 nm, which is consistent with the described UV-Visible spectral region of AuNPs between 500 and 600 nm (Pashkov et al., 2021). XRD configuration endorsing the crystalline features of DR-AuNPs. The FTIR spectrum of *D. retrospiciens* leaf extract shows the occurrence of primary phytochemicals, such as carbohydrates, steroids, tannins, alkaloids, flavonoids, and saponins as described by others (Shahat et al., 2005; Madouh and Davidson, 2024). Furthermore, the measurement of the zeta potential of the phyto-fabricated DR-AuNPs proposed that these nanoparticles had higher stability. The EDX examination of DR-AuNPs revealed an optical absorption band at 2.2 keV, which is the distinctive absorption of metal AuNPs (Elavazhagan and Arunachalam, 2011).

Our *in vitro* data confirmed the antibacterial activity of DR-AuNPs against MRSA-induced osteomyelitis. Several studies have reported that plant-based AuNPs can significantly inhibit the growth of MRSA *in vitro*. For example, phyto-synthesized AuNPs extracts of *Plinia cauliflora, Punica granatum*, and *Hippeastrum hybridum* displayed powerful antibacterial activity against MRSA (Franzolin et al., 2022; Sher et al., 2022).

Several studies have described the design of nano-composition for the treatment of MRSA-induced osteomyelitis with antibacterial and osteogenic capacities. For example, mesoporous silica nanoparticles are reported to reduce the *S. aureus* biomass, impair biofilm development, and significantly decrease the infection in an *in vitro* osteomyelitis model (Aguilera-Correa et al., 2022). Additionally, AgNPs-coated stainless steel nails and copper sericin metal–organic frameworks are reported to exert therapeutic effect against *S. aureus*—prompted osteomyelitis (Wang et al., 2024; Kundu et al., 2022).

The antibacterial action of DR-AuNPs against MRSA might be attributed to the role of DR-AuNPs as a vehicle for plant phytochemicals, which act as capping and stabilizing agents, supporting the dissemination into MRSA cells. AuNPs adhere to the MRSA surface and alter the membrane structural integrity. Finally, nanoparticles penetrate the MRSA cell and interact with its intracellular constituents, damaging it until it cannot perform active cellular processes (Lee and Jun, 2019). Additionally, the alteration of vital signaling transduction, which is essential for the bacterial life cycle, is one of the mechanisms of action of nanoparticles (Masimen et al., 2022). Furthermore, consistent with previously reported data, DR-AuNPs were shown to impair the outer cell membrane of MRSA and thus cause bacterial death (Huynh et al., 2023).

Our data demonstrated the capacity of DR-AuNPs to significantly increase the intracellular ROS production in bacterial cells. Similarly, AuNPs reported to improve the bactericidal activity of methylene blue on MRSA via increasing the accumulation of ROS (Perni et al., 2009). In addition, Au-NPs were shown to induce ROS and to produce free radicals, thus activating irreversible oxidative damage to the bacteria (Durán et al., 2016).

Exploring the anti-biofilm activity of osteomyelitis-infection strains is important for clinicians to understand, assess, and control biofilm-associated orthopedic infections. DR-AuNPs suppressed the *in vitro* biofilm development of MRSA-induced osteomyelitis via inhibiting the surface adherence of MRSA. In this regard, AuNPs are reported to damage the bacterial biofilm to kill bacteria via different mechanisms, which include interfering with the quorum sensing system and reducing the hydrophobicity index (Manju et al., 2016; Ali et al., 2020).

Our data demonstrated the osteoinductive activity of DR-AuNPs in stimulating the early commitment of BMSCs into osteoblast lineage and enhancing their *in vivo* ectopic bone formation capacity. Similarly, plant-based Au-NPs reported to significantly enhance osteogenesis. For example, *Eucalyptus globulus* leaf extract-based Au-NPs and *Panax ginseng* root extract-based Au-NPs were shown to stimulate the osteogenesis of MC3T3-E1 osteoblast-like cells and BMSCs, respectively (Kepekci et al., 2019; Guan et al., 2021). Interestingly, no data available on the *in vivo* effect of green Au-NPs on enhancing bone formation. However, many studies reported promising therapeutic effects of conventionally synthesized Au-NPs in bone tissue regeneration. For example, crude enzyme extracts of *Bacillus licheniformis*-mediated biosynthesized Au-NPs have been shown to facilitate bone regeneration upon integration with scaffold (Singh et al., 2020). A biodegradable hydrogel loaded with Au-NPs demonstrated significant potential in enhancing new bone formation in an *in vivo* bone defect model (Heo et al., 2014). Thus, this is the first report to demonstrate the osteoinductive potential of plant-based Au-NPs in enhancing ectopic bone formation capacity of BMSCs *in vivo*.

Several key signaling pathways are involved in the regulation of osteogenesis, bone formation, and bone homeostasis, including TGF-β/BMP, Wnt, Hedgehog, PTH, and MAPK (Wang et al., 2025; Abdallah et al., 2015). The osteoblast differentiation mechanism is mediated by activating MAPK signaling pathway, which is involved in the activation of the key regulatory factor of osteogenesis, RUNX2, and its downstream-related osteogenic markers (Ge et al., 2016; Abdallah et al., 2015; Wang et al., 2025; Xiao et al., 2000). In this context, we demonstrated that the stimulatory effect of DR-AuNPs on osteoblast differentiation of BMSCs is mediated via activating MAPK ERK1/2. Similarly, citrate-reduced AuNPs are reported to stimulate the bone-derived MSCs osteogenesis via activating p38 MAPK (Yi et al., 2010). On the other hand, chitosan-conjugated AuNPs promote the osteogenesis of adipose-derived MSC via activating the Wnt/β-catenin signaling pathway (Choi et al., 2015). Thus, it appears that the regulatory mechanism of osteogenesis by Au-NPs depends on the fabrication method of nanoparticles and the type of cells used.

## Conclusion

Osteomyelitis is a chronic inflammation of bone caused by a pathogenic infection. We phyto-fabricate AuNPs using plant extract of *D. retrospiciens* (DR-AuNPs) and examined their potential as a green strategic therapy for osteomyelitis. Interestingly, DR-AuNPs exhibited antibacterial activity against MRSA-induced osteomyelitis by increasing ROS production, inducing membrane leakage, membrane permeability, and disrupting the surface structure of MRSA. In addition, DR-AuNPs exhibited a significant osteoinductive effect, promoting the osteoblast differentiation of BMSCs *in vitro* and enhancing their ectopic bone formation capacity *in vivo*. Thus, our data provided green DR-AuNPs as a promising agent for management of osteomyelitis.

## Data Availability

The original contributions presented in the study are included in the article/Supplementary material, further inquiries can be directed to the corresponding author.
