# Supplementary material for: Dual function of Desmidorchis retrospiciens-derived gold nanoparticles as antibacterial and osteoinductive agent for treating osteomyelitis
Source: Front Microbiol. 2025 Aug 4;16:1633245. doi: 10.3389/fmicb.2025.1633245 (PMC12358747; doi:10.3389/fmicb.2025.1633245)
Supplement: Supplementary file 1 [file Data_Sheet_1.PDF]

Supplementary Figure 1

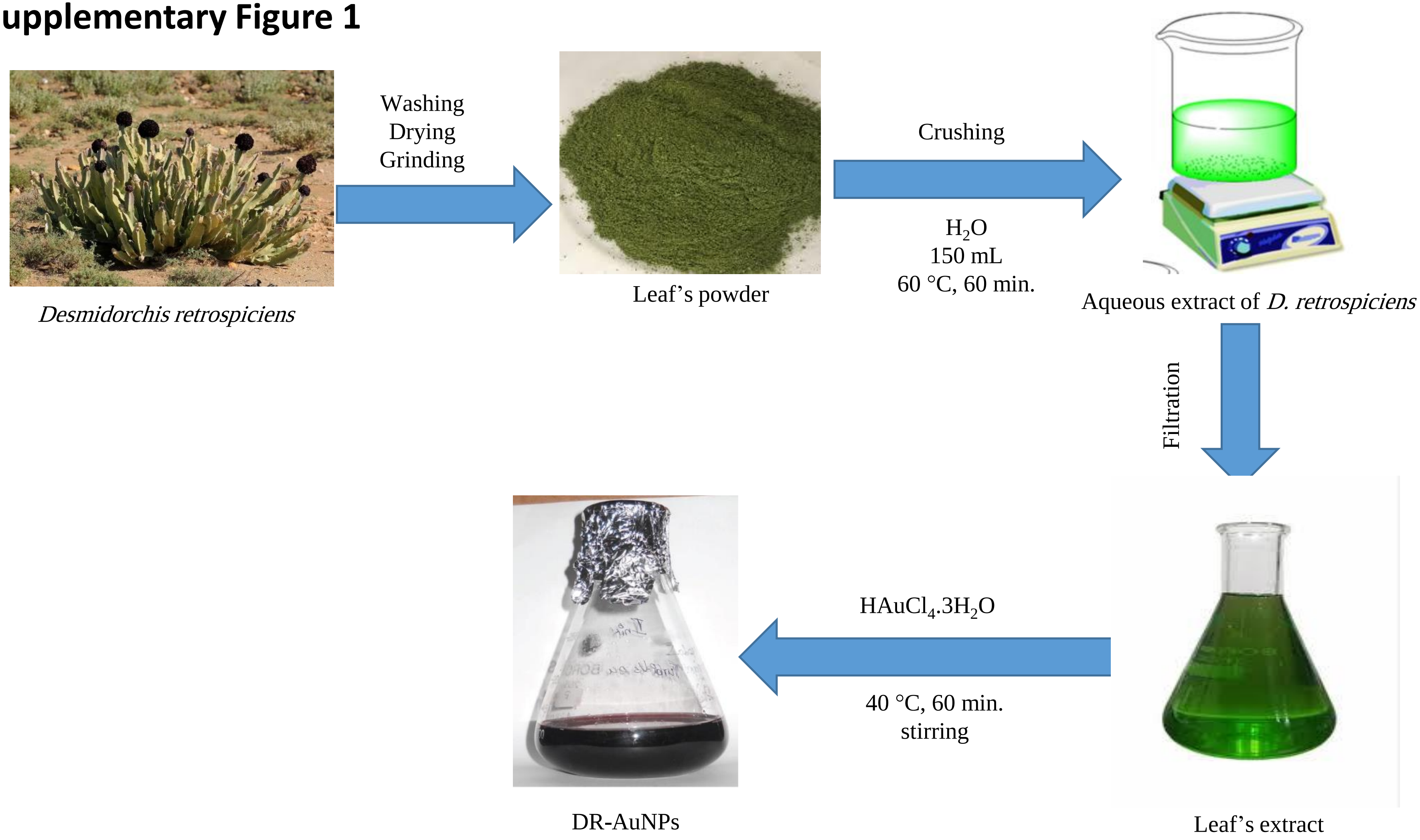

Figure S1. Biosynthesis of DR-AuNPs*.* Color changes from greenish yellow to ruby red designates the reduction of the Au^+ ions and the biosynthesis of AuNPs.
